# Time to re-think the use of dobutamine in sepsis

**DOI:** 10.1186/s40560-017-0264-6

**Published:** 2017-11-21

**Authors:** Ryota Sato, Michitaka Nasu

**Affiliations:** 1 Department of Emergency and Critical Care Medicine, Urasoe General Hospital, Okinawa, Japan; 2 Department of Internal Medicine, John A. Burns School of Medicine, University of Hawaii at Manoa, 1356 Lusitana Street, 7th Floor, Honolulu, HI 96813 USA

**Keywords:** Sepsis, Septic shock, Dobutamine, Inotropes, Sepsis induced cardiomyopathy, Septic cardiomyopathy

## Abstract

Dobutamine is commonly used worldwide and included in the protocol for early goal-directed therapy (EGDT). Since the use of dobutamine in EGDT was reported, it has been considered to be an important component, especially in the treatment of septic patients with myocardial dysfunction. However, it is questionable whether dobutamine improves the mortality of sepsis and septic shock.

In three recent randomized controlled trials (ProCESS, ProMISe, and ARISE trials), the frequency of dobutamine use was significantly higher in the EGDT group than in the standard care group, but there were no significant differences in the mortality between the groups. These results suggested that dobutamine use may have been overemphasized despite its insignificant effect on the mortality in septic patients. Further, a propensity score analysis revealed that dobutamine use was associated with higher mortality in patients with septic shock.

Although dobutamine leads to an increase in cardiac index, myocardial oxygen demand also increases, thus increasing the risk of myocardial ischemia and tachyarrhythmia. It is well known that the mortality in sepsis complicated with atrial fibrillation (AFib) is worse than that in sepsis without AFib. A propensity score-matched analysis reported that β-blockers were associated with better survival in patients with sepsis complicated with AFib. Further, a randomized controlled trial reported that a short-acting β-blocker improved the survival in patients with septic shock. These studies also indicated the risk of β-stimulation during sepsis.

Notably, improvements in surrogate markers, such as CI, do not always indicate improvements in patient-centered outcomes, such as mortality. Conversely, some evidence indicates the worsening of patient-centered outcomes despite improvements in surrogate markers.

Thus, available evidence suggests that the benefits of dobutamine in patients with sepsis are unclear, but its use might be harmful rather than beneficial, considering the beneficial effects of β-blockers in sepsis that have been reported in recent clinical studies. From this perspective, we will soon have to rethink regarding dobutamine use in patients with sepsis.

## Manuscript

Dobutamine is a synthetic catecholamine that acts on α-1, β-1, and β-2 adrenergic receptors. It is commonly used worldwide and included in the protocol for early goal-directed therapy (EGDT) [1]. Since the use of dobutamine in EGDT was reported, it has been considered to be an important component, especially in the treatment of septic patients with myocardial dysfunction. Currently, surviving sepsis campaign guidelines suggest the use of dobutamine in the presence of myocardial dysfunction indicated by elevated cardiac filling pressures and low cardiac output or ongoing signs of hypoperfusion, despite achieving adequate intravascular volume and mean arterial pressure [2]. However, it is questionable whether dobutamine improves the mortality of sepsis and septic shock.

The results of three randomized controlled trials (ProCESS, ProMISe, and ARISE trials) on resuscitation in sepsis have been recently published [3–5]. In these trials, the frequency of dobutamine use was significantly higher in the EGDT group than in the standard care group (ProCESS trial 8.0 vs. 1.1%, respectively; *p* < 0.001; ProMISe trial 8.0 vs. 1.1%, respectively; *p* < 0.001; and ARISE trial 15.4 vs. 2.6%, respectively; *p* < 0.0001), but there were no significant differences in the mortality between the groups. Although recent meta-analysis of randomized trials suggested inodilators such as levosimendan and dobutamine might improve the survival in septic patients [6], most studies comparing inotropes included in this study were small single center studies, and we have to be careful to interpret the results. Furthermore, the biggest randomized controlled trial showed no benefit of levosimendan in mortality or prevention for organ dysfunction in septic patients [7]. These results suggested that use of inotropes may have been overemphasized despite its insignificant effect on the mortality in patients with sepsis. Further, a propensity score analysis revealed that dobutamine use was associated with higher mortality in patients with septic shock [8].

Conversely, in patients with low cardiac index (CI), the benefit of dobutamine use continues to be unclear. Vieillard-Baron et al. reported simultaneous Doppler echocardiographic measurement of CI and left ventricular ejection fraction (LVEF) in patients with septic shock and estimated the association between them using the following formula: CI = (0.05 × LVEF) + 0.73 [9]. According to this formula, LVEF of 35% is associated with a CI of 2.5, which is the lower threshold of the normal CI range. Because maintaining supranormal CI using dobutamine is not associated with better survival [10], LVEF of 35%, but not < 35%, may be sufficient, and a relatively low LVEF (35–50%) may not be associated with mortality. The population with LVEF < 35% (CI < 2.5) represents a small percentage of patients with septic shock and may be more likely to benefit from dobutamine use. However, a meta-analysis reported that dobutamine did not improve the mortality in patients with severe heart failure, both in outpatient and inpatient settings [11]. In this study, mean CI ranged from 1.7 to 2.5 and mean LVEF from 20 to 35%, suggesting that dobutamine use may not be effective even in patients with low CI. Since β-1 receptor is known to be downregulated in patients with heart failure [12], we have to be careful to interpret this result. However, even among septic patients, myocardial adrenergic responsiveness is considered to be depressed [13]. Therefore, the use of dobutamine in septic patients may not be effective as well as patients with severe heart failure.

Further, previous randomized control trials reported that maintaining higher CI than normal with dobutamine did not affect mortality while dobutamine successfully elevated CI even in patients with normal CI [10, 14]. Although dobutamine leads to an increase in CI and splanchnic blood flow [15], myocardial oxygen demand also increases, thus increasing the risk of myocardial ischemia and tachyarrhythmia [16]. It is well known that the mortality in sepsis complicated with atrial fibrillation (AFib) is worse than that in sepsis without AFib [17]. A propensity score-matched analysis reported that β-blockers were associated with better survival in patients with sepsis complicated with AFib [18]. Further, a randomized controlled trial reported that a short-acting β-blocker improved the survival in patients with septic shock [19]. These studies also indicated the risk of β-stimulation during sepsis.

Notably, improvements in surrogate markers, such as CI, do not always indicate improvements in patient-centered outcomes, such as mortality. Conversely, some evidence indicates the worsening of patient-centered outcomes despite improvements in surrogate markers.

Thus, available evidence suggests that the benefits of dobutamine in patients with sepsis are unclear, but its use might be harmful rather than beneficial, considering the beneficial effects of β-blockers in sepsis that have been reported in recent clinical studies. From this perspective, we will soon have to rethink regarding dobutamine use in patients with sepsis.

## Abbreviations

AFib: Atrial fibrillation
CI: Cardiac index
EGDT: Early goal-directed therapy
LVEF: Left ventricular ejection fraction
